# Three-Dimensional Metallic Surface Micropatterning through Tailored Photolithography–Transfer–Plating

**DOI:** 10.1021/acsami.4c10550

**Published:** 2024-08-20

**Authors:** Liyang Chen, Julian Schmid, Anetta Platek-Mielczarek, Tobias Armstrong, Thomas M. Schutzius

**Affiliations:** † Laboratory for Multiphase Thermofluidics and Surface Nanoengineering, Department of Mechanical and Process Engineering, ETH Zurich, Sonneggstrasse 3, CH-8092 Zurich, Switzerland; ‡ Department of Mechanical Engineering, University of California, Berkeley, Berkeley, California 94720, United States

**Keywords:** precision micropatterning, surface engineering, 3D micropatterning, robust metallic microstructures, transfer printing, plated microstructures

## Abstract

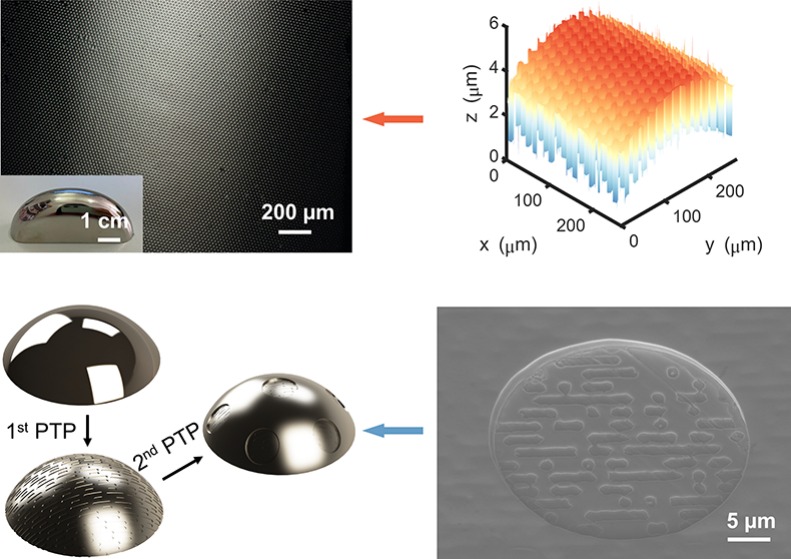

Precise micropatterning on three-dimensional (3D) surfaces is desired for a variety of applications, from microelectronics to metamaterials, which can be realized by transfer printing techniques. However, a nontrivial deficiency of this approach is that the transferred microstructures are adsorbed on the target surface with weak adhesion, limiting the applications to external force-free conditions. We propose a scalable “photolithography–transfer–plating” method to pattern stable and durable microstructures on 3D metallic surfaces with precise dimension and location control of the micropatterns. Surface patterning on metallic parts with different metals and isotropic and anisotropic curvatures is showcased. This method can also fabricate hierarchical structures with nanoscale vertical and microscale horizontal dimensions. The plated patterns are stable enough to mold soft materials, and the structure durability is validated by 24 h thermofluidic tests. We demonstrate micropatterned nickel electrodes for oxygen evolution reaction acceleration in hydrogen production, showing the potential of micropatterned 3D metallic surfaces for energy applications.

## Introduction

Precision surface engineering of metallic surfaces with stable and durable micropatterns is desired for applications involving biomedical engineering, tribology, thermal management, and renewable energy. For instance, anti-biofouling micropatterns can reduce foreign body reaction risks from implanted devices;^1−3^ rational modification of the solid/gas interface by micropatterning facilitates wicking and lubrication, as in pins and thrust bearings;^4−7^ surface micropatterning can modify heat transfer coefficients (HTCs) to accelerate heat dissipation for vapor chambers^8−10^ or reduce heat loss in domestic heat supply systems;^11,12^ and micropatterns on the electrodes of alkaline water electrolyzers (AWEs) can help release hydrogen and oxygen bubbles and thus improve cell efficiencies.^13−17^

Despite the great demand for precise and robust micropatterning on three-dimensional (3D) metallic surfaces, existing manufacturing techniques cannot meet the needs. Laser ablation is a straightforward approach for micropatterning metallic surfaces.^18,19^ However, the topography of the written microstructures is rather rough due to the uneven spatial power distribution of the laser source.^20−22^ Transfer printing techniques are well developed to micropattern 3D surfaces with high precision^23,24^ and scalability,^25−27^ which consist of pattern formation on planar substrates and pattern transfer to curved substrates with carriers.^28−33^ However, metallic microstructures can be transferred to curved dielectric substrates via electrostatic adhesion from dielectric polarization^26^ but not to metallic surfaces. Microcontact printing that transfers self-assembled monolayers (SAMs) as etching or deposition masks was the only feasible solution to precisely micropattern curved metallic substrates. But this method relying on the strong sulfur–metal bonds does not apply to Ni—the most popular surface finishing and mold material due to its excellent resistance to oxidation and corrosion.^34^ Moreover, microcontact printing normally generates microstructures thinner than 100 nm because the SAM masks are unstable for deep etching and too thin for thick deposition.^35,36^

Here we propose a “photolithography–transfer–plating” (PTP) method to fill the gap of patterning well-defined micrometer thick structures on curved metallic substrates. This method combines the advantages of precise large-area surface micropatterning from transfer printing techniques and excellent structure stability and durability from plating techniques to implement surface engineering on 3D metallic objects. We characterized the topography of micropatterned 3D metallic surfaces through optical microscopy, white light interferometry (WLI), and scanning electron microscopy (SEM), demonstrating high precision patterning with nanoscale vertical and horizontal dimensions. The stability of the plated structure is validated by soft lithography, where the surface structure on a patterned 3D metallic part is successfully transferred to polydimethylsiloxane (PDMS). The durability of the metallic surface pattern is confirmed by a 24 h fluidic test, where no deformation or delamination is observed. To stress the great potential of employing surface micropatterned 3D metallic parts for energy applications, we micropatterned nickel electrode surfaces in an AWE to accelerate oxygen bubble release and thus to improve the oxygen evolution reaction (OER) and water electrolysis efficiencies.

## Results and Discussion

### Large-Area Precise Micropatterning on 3D Metallic Surfaces

**Figure 1:**
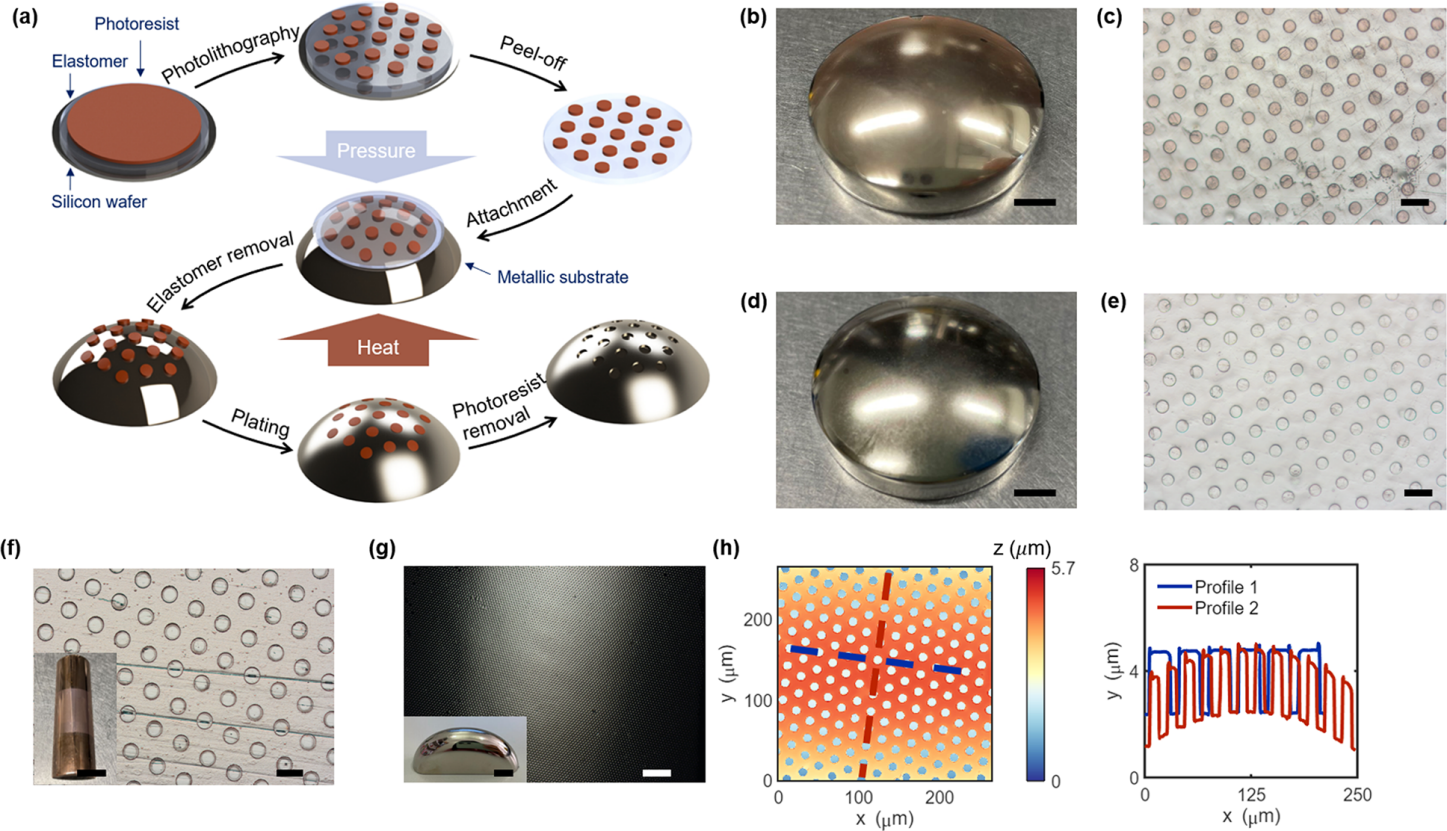
Fabrication and characterization of micropatterned 3D metallic surfaces. (a) Fabrication schematic of the PTP method for patterning 3D metallic surfaces. (b) Digital photo and (c) optical microscopic images of transferred photoresist micropillars on a spherical stainless steel surface. The lines in (c) are scratches on the substrate existing before the photoresist transfer. (d) Digital photo and (e) optical microscopic image of the 3D stainless steel surface with electroless plated nickel microholes. (f) A copper half tube with an outer diameter of 14 mm patterned with 60 μm pitch and 30 μm diameter microholes. (g) A stainless steel sample with anisotropic curvatures patterned with 20 μm pitch and 10 μm diameter microholes. (h) is the surface profile of (g) acquired by WLI. Scale bars: (b, d) 5 mm; (c, e) 20 μm; (f) 50 μm; (g) 200 μm; insets in (f, g) 1 cm.

Figure 1a illustrates the fabrication schematic of the PTP method to pattern 3D metallic surfaces. Photoresist micropillars are fabricated by photolithography on thin PDMS films coated on Si wafers. The PDMS film can be easily peeled off from the Si wafer that is pretreated by dichlorodimethylsilane with a hydrophobic functional group. With the flexible and stretchable PDMS film conformally wrapping curved metallic surfaces, thermoplastic photoresist micropillars can be transferred under heat and pressure. Since the adhesion of the photoresist microstructure to the metallic surface is better than that to the PDMS, photoresist patterns are left on the metallic part when the PDMS film is removed, which are subsequently used as plating masks for depositing metals on the substrate and are washed off by acetone after the plating process, leaving metallic microhole structures on the 3D metallic surface.

The PTP method proves to be a robust process due to excellent transfer yields and stability (as plating masks) of the photoresist patterns. Figures 1b and 1c show a near 100% transfer yield on a spherical stainless steel substrate with photoresist micropillars of 10 μm in diameter. The adhesion between the transferred photoresist and metallic substrates is strong enough to resist the disturbance of gas bubbles generated during the plating processes. Figures 1d and 1e demonstrate successful metallization of the micropatterns on stainless steel surface with electroless plated nickel. Electroless plating features uniform deposition on curved surfaces that is hard to achieve by electroplating, and the thickness of the structures can be precisely controlled from nanometers to microns by adjusting the plating parameter.^37^ It is worth noting that the feature size of pits on the substrate should be smaller than the photoresist pillar diameter with the demonstrated setup because the micropillars cannot be transferred into the pit (Figure S1), but this challenge can be solved using a setup with a dedicated pressure supply.

The PTP method can be applied to diverse metallic material systems and substrates with various curvatures. The material selection is application-oriented but restrained by options for plating.^38^ Nickel and copper are selected for our demonstration due to their wide applications in daily life: nickel is the most commonly used material for stainless steel coatings due to its excellent corrosion resistance and good electrical conductivity;^39^ copper possesses the highest thermal conductivity among non-noble metals and is intensively used for heat transfer-related applications.^40^Figures 1f and 1g validate the capability of the PTP method to pattern a cylindrical copper substrate and a complex nickel substrate with precise thickness control, respectively. Figure 1h plots the topography of the micropatterned 3D surface in Figure 1g and its cross-section profiles. The surface profile declares that the plated microstructure is conformally grown on the 3D substrate with superfine grains that cannot even be differentiated by WLI. It is worth noting that the PTP method manages to pattern microstructures on both convex and concave surfaces, as demonstrated on a wavy substrate (Figure S2), where both the peak and the valley are structured. A complementary part of the wavy substrate is needed to transfer photoresist micropillars to concave surfaces.

While the PTP method is capable of patterning microstructures on metallic substrates of different materials and with various curvatures, there are some limitations and aspects to improve. First of all, the PTP method is unable to fabricate protruding metallic features because the patterned photoresist film cracks when stretched due to its rigidity (Figure S3). This limitation can be resolved by using some novel elastic photoresists,^41^ although they are not yet commercially available. Another concern is the inevitable deformation of the micropatterns after transfer with stretching of the elastomer. This challenge can be potentially addressed by the computation-assisted 3D reconstruction algorithm, which is able to calculate accurate 2D patterns for desired projection patterns on 3D objects.^42^ With this algorithm, tailored micropatterns on 3D metallic substrates can be achieved via the rational design of 2D microstructures on photolithography masks.

### Hierarchical Microstructures

**Figure 2:**
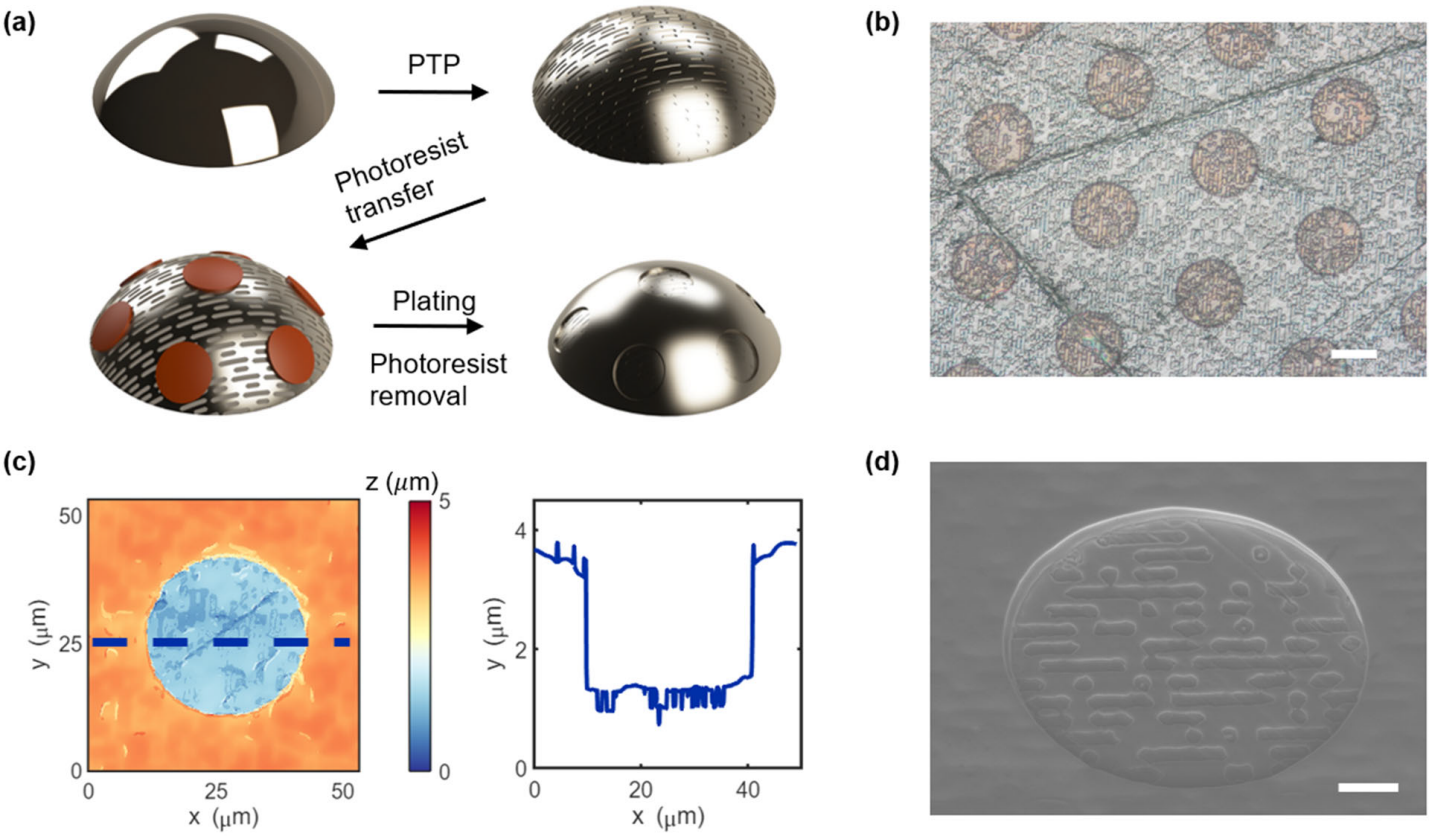
Fabrication and characterization of hierarchical microstructures. (a) Fabrication schematic of patterning hierarchical structures on 3D metallic surfaces. (b) Optical microscopic image of transferred photoresist micropillars on prepatterned metallic surface with a finer structure. (c) Surface profile of the hierarchical nickel structure acquired by WLI. (d) A 40° tilted top-view SEM image of the hierarchical nickel structure. Scale bars: (b) 20 μm and (d) 5 μm.

Hierarchical microstructures can push the surface properties to extreme conditions where a monoscale pattern can hardly be achieved.^43,44^ For instance, superoleophobic surfaces are obtained on hierarchical metal meshes,^45^ and superhydrophilic anti-biofouling metallic surfaces are realized with hierarchical topography.^46^ So far, well-defined hierarchical microstructures—excluding random surface structures synthesized by chemical reactions—can be patterned only on planar metallic surfaces. Our PTP method can overcome this challenge by process repetition. Figure 2a shows the fabrication schematic of patterning 3D metallic surfaces with hierarchical structures. The metallic substrate is first patterned with a smaller structure; then, the PTP process is repeated to add on a larger feature size. Hierarchical structures with even more levels are theoretically achievable with multiple PTP process repetition. Here we demonstrate two-level hierarchical patterning on a stainless steel substrate. We successfully transferred photoresist microdisks with a diameter of 30 μm (Figure 2b) to the surface prepatterned with 300 nm thick wire/dot structures. After metallization of the larger structure and removal of the photoresist mask, hierarchical nickel microstructures are fabricated; Figure 2c depicts the topographic information on the metallic hierarchical structure from WLI, showing the nanometer thick structures on the bottom of a microhole. Figure 2d demonstrates the morphology of the hierarchical structure obtained from SEM.

### Stability and Durability Validation

**Figure 3:**
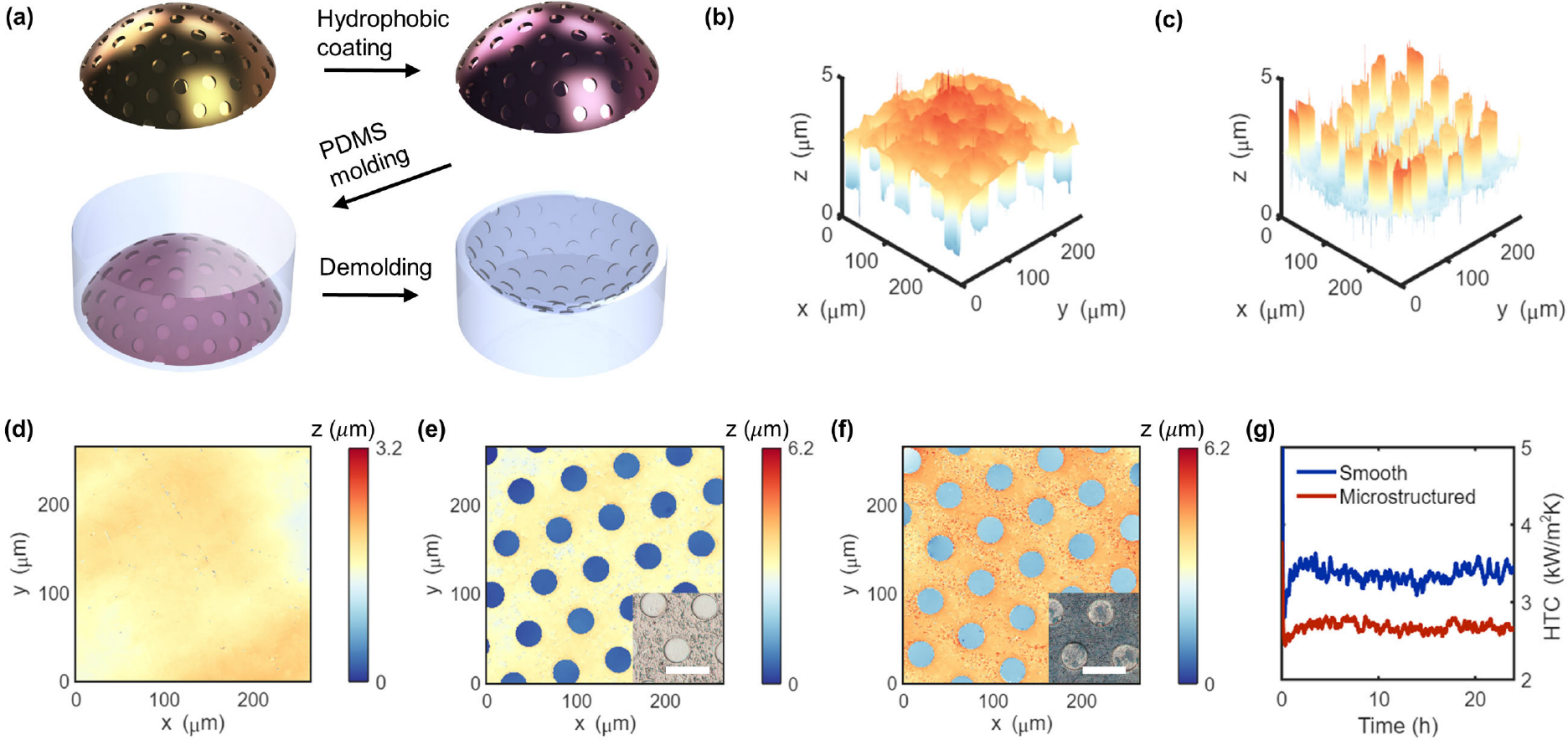
Stability and durability tests of the microstructures on curved metallic surfaces. (a) Fabrication schematic of pattern transfer from micropatterned metallic molds to soft materials. 3D mapping of the topography of (b) a metallic mold and (c) the PDMS replicate obtained by WLI. Surface profiles of (d) smooth and (e) microstructured copper surfaces before fluidic tests. (f) Topography of the micropatterned copper surface after a 24 h fluidic test. The insets in (e) and (f) are optical microscopic images of the micropatterned copper surface before and after the fluidic test, respectively. (g) Real-time measurement of HTCs of smooth and micropatterned copper surfaces during 24 h fluidic tests. A moving mean of local 61 points (600 s) is calculated and plotted. The uncertainty of the HTC measurement is 1.3%. Scale bars: 50 μm.

We validate the stability of the metallic microstructures fabricated via the PTP method by transferring the surface pattern to PDMS via soft lithography. Surface micropatterning on 3D soft materials has tremendous value in applications such as self-cleaning flexible wearable sensors,^47^ antioxidant skin patches,^48^ antibacterial implants,^49,50^ and drag reduction for underwater robots.^51^Figure 3a illustrates the schematic of patterning PDMS bulk material with microstructures using a patterned metallic mold. The mold can be made of various metals, such as stainless steel, nickel, and copper. A thin layer of copper is deposited on the surface of the mold if it is non-copper material, which is decorated with hydrophobic coating to facilitate the demolding process. Figures 3b and 3c demonstrate the complementary topography of a spherical metallic mold and the corresponding PDMS replicate, respectively. They show successful pattern transfer with soft lithography, which is scalable to industrial applications due to its compatibility with injection molding.

We conducted 24 h thermofluidic tests to validate the durability of the metallic surface micropatterns fabricated by the PTP method, which verifies the possibility of endowing 3D metallic objects with durable surface modification for thermal management such as anti-icing^52−55^ and phase change heat transfer.^56−58^ Here we patterned a smooth copper surface with microhole structures (60 μm in pitch, 30 μm in diameter, and 2 μm in depth). Figures 3d and 3e display the topography of a smooth copper surface and the micropatterned one, respectively. Both samples were placed in a homemade fluidic system for 24 h real-time characterization of the stability of the surface pattern under realistic heat transfer conditions. The plated microstructure is robust enough to endure continuous water flushing and heating for 24 h without pattern removal, as evidenced in Figure 3f, which depicts the morphology of the micropatterned sample after the fluidic test. Increased surface roughness after the test is visible, which is supposed to be attributed to surface copper oxidation. The optical microscopic images (insets in Figures 3e and 3f) of the micropatterned copper surface before and after the fluidic test clearly show the oxidation-resulting color change of the copper surface. The same change also happens to the smooth sample (Figure S4). HTCs of both the smooth and micropatterned copper samples were monitored and are presented in Figure 3g. The stable HTC of the patterned sample indicates intact micropatterns through the course of the 24 h fluidic test.

### Metal Surface Patterning for Hydrogen Production

**Figure 4:**
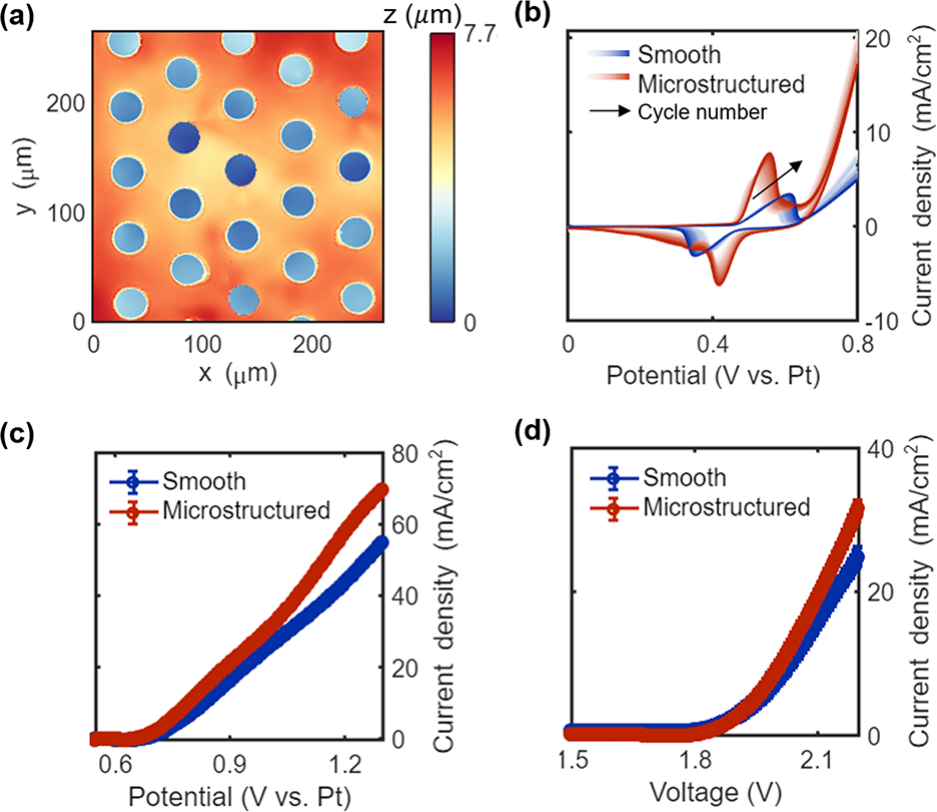
Surface microstructure-facilitated water electrolysis. (a) Surface profile of a microstructured nickel electrode of an AWE acquired by WLI. Comparison of (b) 200 CV cycles at 50 mV/s and (c) OER tests with a scan rate of 5 mV/s of smooth and micropatterned nickel electrodes. Error bar calculated from *n* = 3 times experiments for each electrode. (d) Water splitting tests of AWEs composed of smooth and microstructured electrodes. Error bar calculated from *n* = 3 times experiments for each electrolyzer.

The PTP method can benefit green hydrogen production by optimizing the electrode design for higher cell efficiencies. One of the biggest obstacles to improving AWE efficiencies is the difficulty in removing gas bubbles generated on the electrode surface. Quick removal of gas bubbles is needed as the bubble on the electrode surface blocks reaction sites and thus lowers the cell efficiency,^59^ which usually requires an external water circulation system increasing energy input.^60^ Researchers have already proved that microhole structures on the nickel electrode surface are effective in accelerating oxygen bubble removal as well as the OER.^61^ On the other hand, nonplanar electrodes are reported to have higher power efficiency and hydrogen production rate than plate electrodes.^62^ Therefore, nonplanar electrodes with surface micropatterning are supposed to be the optimal design of nickel electrodes for AWEs. Here we compared the electrochemical properties of smooth and micropatterned nickel electrodes with curved surfaces, and the results reveal improved performance from surface micropatterning in the OER process. Figure 4a depicts the topography of a nickel electrode with surface micropatterns (4 μm depth, 60 μm pitch, and 30 μm diameter). Cyclic voltammetry (CV) was applied to the smooth and micropatterned electrodes in 1 M KOH solution for sample conditioning to obtain a uniform nickel phase. Figure 4b plots 200 cycle profiles at a scan rate of 50 mV/s, showing a clear shift in the position of nickel oxidation and reduction peaks of both smooth and micropatterned electrodes. The CV finally stabilized, indicating no further changes of sample’s surface. Figure 4c compares the OER activity of the smooth and micropatterned electrodes assessed by linear sweep voltammetry at 5 mV/s in the 1 M KOH solution. A slightly lower overpotential and an increase in current density of the micropatterned electrode are observed, indicating improved OER activity due to surface patterning.^4^ The current density of the micropatterned electrode is calculated using the specific surface area, which is 1.121 times the geometric surface area of the smooth sample. Consequently, improved water splitting performance indicated by a higher current density of the electrolyzer is achieved with the introduction of micropatterns, as shown in Figure 4d. In both 3-electrode and 2-electrode setups, gas evolution was uniform, and gas bubbles did not accumulate on the micropatterned surface in comparison to the smooth one. Note that the noticeable performance improvement is realized without microstructure optimization for this specific application, which suggests the promise of a further boost in AWE efficiencies with parameter optimization.

## Conclusions

In conclusion, we developed the PTP method to pattern large-area microstructures on 3D metallic surfaces. This method enables precise surface patterning by combining accurate positioning from transfer printing techniques and fine pattern thickness control from electro-/electroless plating. Excellent stability of the plated surface structure enables micropatterned 3D topography transfer from metallic parts to soft materials by soft lithography, and the durability of the plated structure is verified by 24 h fluidic tests. The limitations of the PTP method were also discussed with promising potential solutions from material innovation and a computation-assisted 3D reconstruction algorithm. We finally demonstrated the great potential of employing micropatterned metallic surfaces for energy applications such as improving the OER efficiency and thus the water electrolysis efficiency of green hydrogen production.

## Methods

### Micropatterning on 3D Metallic Surfaces

#### Photolithography

The 4 in. Si wafers are silanized by immersing the wafer in silanization solution I (Sigma-Aldrich) for 30 min, followed by rinse with ethanol and dried with nitrogen; then the wafer is baked at 60 °C for 60 min on a hot plate, followed by consecutive rinse with ethanol and deionized water and dried with nitrogen. PDMS (Sylgard 184) is synthesized by mixing the base and curing agent with a weight ratio of 10:1, and the mixture is degassed in a vacuum chamber for 30 min. 2.5 g of PDMS is added on the silanized wafer and spun at 400 rpm for 30 s, which is baked at 80 °C for 2 h for curing. Positive photoresists MICROPOST S1813 and S1805 are spin-coated on the PDMS film with various spin-coating speeds for different thicknesses and baked at 115 °C for 5 min. Photolithography is done using the BA/BA6 mask aligner (SUSS MICROTEC) with various exposure doses according to the photoresist thickness, and the development is done with a MICROPOST MIF-319 developer.

#### Pattern Transfer

Metallic (stainless steel and copper) substrates were polished by Ateco Tobler AG. The polished substrates were cleaned by ultrasonication with acetone, isopropanol, and deionized water for 5 min each. The photoresist micropattern-coated PDMS film is peeled off from the wafer and fixed on a ring. Metallic substrates are placed on a hot plate at 85 °C for 2 h with photoresist-coated PDMS films pressed onto the 3D surface at a pressure of around 1 kPa. Then PDMS films are removed, and the photoresist micropattern is left on the 3D metallic surface.

#### Metal Plating

Metallic microstructures are deposited on the substrate with the photoresist micropattern mask via electroplating or electroless plating. Nickel electroplating and electroless plating are completed with a CASWELL Nickel Electroplating Kit and a CASWELL Electroless Nickel Plating Kit, respectively. Copper is plated using a CASWELL Flash Copper Plating Kit. Plating parameters refer to the CASWELL Plating Manual.

### Soft Lithography of PDMS

The micropatterned nonplanar substrate was first coated with a thin layer of copper by electroplating if it is another material. The substrate becomes hydrophobic by immersing it in 5 mM 1-octadecanethiol (98%, Sigma-Aldrich) in ethanol for 24 h. After that, the substrate was rinsed with ethanol and dried with nitrogen. The substrate is placed in a glass Petri dish for soft lithography. The same PDMS as described previously was poured into the Petri dish and degassed in a vacuum chamber for 2 h. The PDMS was then cured at 80 °C for 6 h, and the microstructures were successfully transferred to a 3D PDMS surface.

### Topographical Characterization

Top-view morphological characterization of transferred photoresist micropatterns and plated metallic micropatterns is done with an optical microscope (Nikon) with magnification of 5×, 20×, and 50×. The topographical information on patterned 3D surfaces is acquired by a white light interferometer (ZYGO) with magnification of 5×, 20×, and 100×, and the data files are imported to MATLAB R2022a and visualized in the format of 3D mapping. High-magnification surface morphology characterization of the hierarchical structure is accomplished with a FEI Nova NanoSEM 450 scanning electron microscope.

### Fluidic Tests

The closed loop fluidic heat transfer setup consists of a membrane pump (Shurflo 8000-543-290), which we connect in series to a flowmeter (BIOTECH, fch-m-pom; accuracy: ±2%), a home-built parallel plate chamber, and a temperature-controlled reservoir. We apply for the 24 h tests flow rates of *V̇* ≈ 2 L/min, resulting in Reynolds number of *Re* ≈ 2500 in the chamber (width 25.1 mm × height 5 mm). The heating zone consists of an external powered heating cartridge (PROBAG HS 208) which we connect to an external power source, an intermediate copper block, and the sample to study. The adjustable heater cartridge maintains a consistently defined power level (±2%) throughout the experiment, set at 20 W for the entire duration of the tests. We ensured uniform temperature distribution by attaching the embedded sample to an intermediate copper block, promoting consistent heat transfer. Thermal paste (Thermal Grizzly Kryonaut Extreme, *k* = 14.2 W/(m K)) enhances conductivity between the sample and the copper block. We use five previously calibrated thermocouples (T-Type, ±0.1 K, *d* = 1 mm) embedded in the copper block with axially uniform spacing to determine the heat flux *q*″ (±5%) using Fourier’s law of conduction, which facilitates the determination of the sample temperature *T*_surf_ by a linear extrapolation. We calculate HTC values at 0.1 Hz using Newton’s law of cooling. The uncertainty of the HTC measurement is 1.3%. We obtained all sensor control and data acquisition using a data acquisition system (Beckhoff) and LabView.

### Characterization of AWEs

Electrochemical characterization of nickel electrodes for the OER process of AWEs is implemented with a potentiostat SP-200 (Biologic, France). All electrochemical tests are done in excess of 1 M KOH aqueous electrolyte. Pt wires are used as both the counter electrode and the quasi-reference electrode of the three-electrode system; a smooth or micropatterned nickel sample is the working electrode. Sample conditioning is done within 250 cycles of CV with a scan rate of 50 mV/s, and the 50th to 250th cycles are plotted and compared as the curves gradually stabilize after the first 50 cycles. OER tests are repeated 3 times for each sample with a linear voltammetry from 0.5 to 1.4 V with a scan rate of 5 mV/s. Water splitting tests of AWEs comprising smooth and micropatterned Ni counter and working electrodes are conducted with a two-electrode configuration for 3 times for each cell with a linear voltage scan from 0.9 to 2.2 V with a scan rate of 5 mV/s.

## Data Availability

All data needed to evaluate the conclusions in the paper are present in the paper and/or the Supporting Information.

## Supporting Information Available

Figure S1: optical microscopic image of photoresist micropillars transferred to unpolished convex metallic surfaces; Figure S2: transferring photoresist micropillar to wavy substrates with concave surfaces; Figure S3: cracked microhole photoresist layer on PDMS film after peeling off from the wafer; Figure S4: comparison of the topography of a smooth copper surface before and after the fluidic test (PDF)
