# Supplementary material for: Three-Dimensional Metallic Surface Micropatterning through Tailored Photolithography–Transfer–Plating
Source: ACS Appl Mater Interfaces. 2024 Aug 20;16(35):46937–44. doi: 10.1021/acsami.4c10550 (PMC11378153; doi:10.1021/acsami.4c10550)
Supplement: Supplementary file 1 — am4c10550_si_001.pdf [file am4c10550_si_001.pdf]

Supporting Information

Three-dimensional metallic surface micropatterning through tailored photolithography-transfer-plating

*Liyang Chen[1], Julian Schmid[1], Anetta Platek-Mielczarek[1], Tobias Armstrong[1], Thomas M. Schutzius[1, 2,] **

[1]Laboratory for Multiphase Thermofluidics and Surface Nanoengineering, Department of Mechanical and Process Engineering, ETH Zurich, Sonneggstrasse 3, CH-8092 Zurich, Switzerland

[2]Department of Mechanical Engineering, University of California, Berkeley, CA 94720, USA

*Email: tschutzius@berkeley.edu

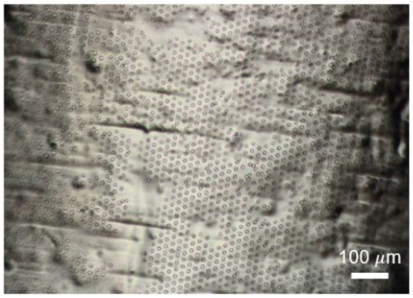

Figure S1. Optical microscopic image of photoresist micropillars transferred unpolished cylindrical stainless-steel surfaces with lots of pits.

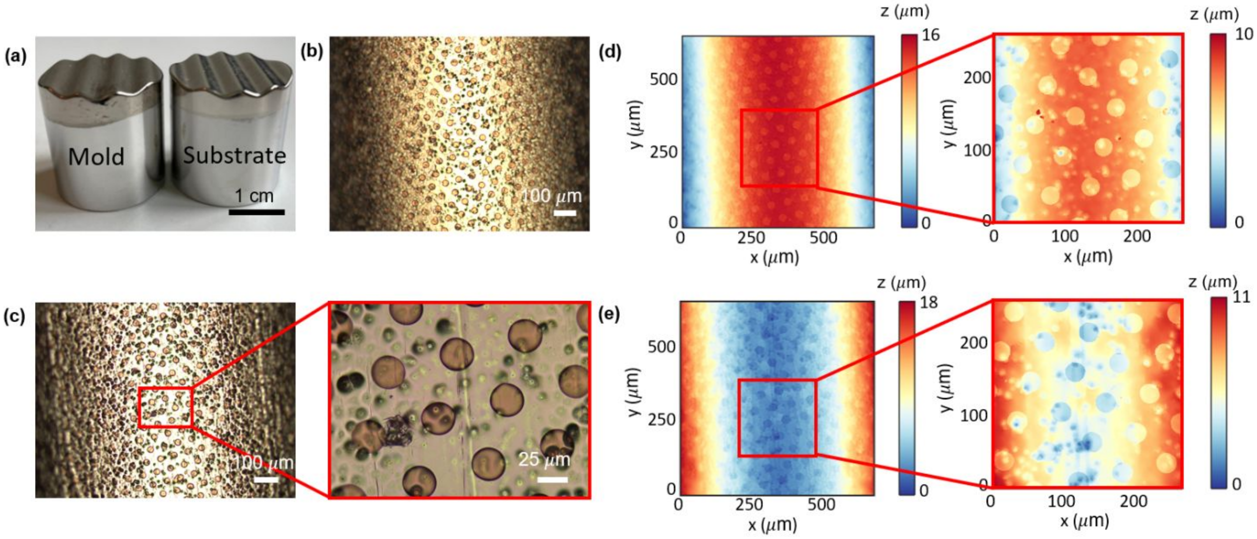

Figure S2. Micropatterning on wavy substrate. (a) A digital photo of a stainless steel substrate with a wavy top surface and a mold with a complementary surface to the substrate. The PDMS carrier with photoresist micropatterns is sandwiched between the substrate and the mold to ensure that the PDMS film is conformally in contact with the wany surface of the substrate. Optical microscopic images of (b) the peak and (c) the valley of the substrate after photoresist transfer. Surface profiles of (d) the peak and (e) the valley of the micropatterned substrate after Ni electroless plating are obtained by WLI. Note that neither optical microscopy nor WLI is able to capture the profile of the steep side wall of the wavy surface as the reflected light cannot be received by the lens.

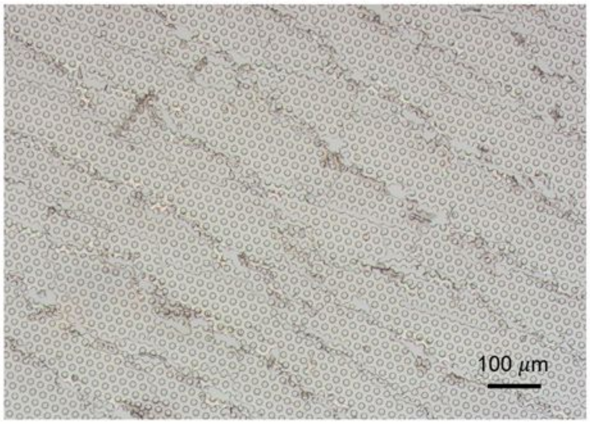

Figure S3. Cracked microhole photoresist layer on PDMS film after peeling off from the wafer.

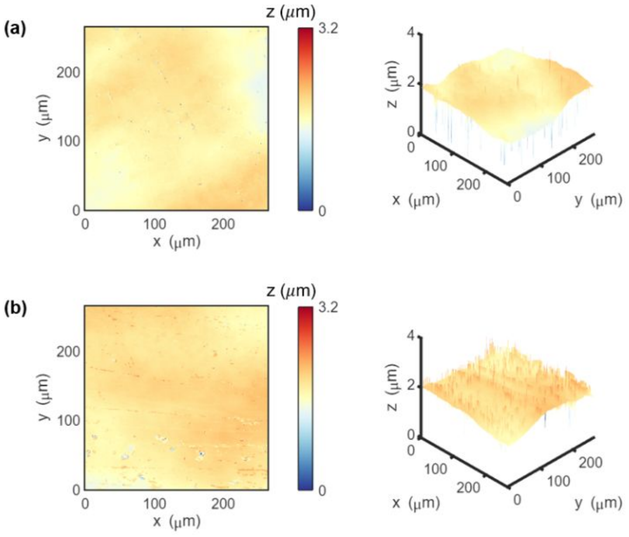

Figure S4. Compared with (a) the fresh smooth sample, the surface roughness of (b) the sample after the fluidic test increased. Topographical mapping acquired by white light interferometry.
